# Male reproductive health challenges: appraisal of wives coping strategies

**DOI:** 10.1186/s12978-017-0341-2

**Published:** 2017-07-28

**Authors:** Emmanuel O. Amoo, Adekunbi K. Omideyi, Theophilus O. Fadayomi, Mofoluwake P. Ajayi, Gbolahan A. Oni, Adenike E. Idowu

**Affiliations:** 1 Andrew Mellon Foundation Postdoctoral Fellow, Demography and Population Studies, Schools of Public Health and Social Sciences, Faculties of Health Sciences and Humanities, University of the Witwatersrand, Johannesburg, South Africa; 2 Department of Demography and Social Statistics, College of Business and Social Sciences, Covenant University, Ota, Ogun State Nigeria; 3 Department of Demography and Social Statistics, Obafemi Awolowo University, Ile Ife, Nigeria; 4 Economics Department, Elizade University, Ilaramokin, Ekiti State Nigeria; 5 Sociology Department, College of Business and Social Sciences, Covenant University, Ota, Ogun State Nigeria; 6 Postdoctoral Fellow, DST-NRF Centre of Excellence in Human Development, Demography and Population Studies, Schools of Public Health and Social Sciences, Faculties of Health Sciences and Humanities, University of the Witwatersrand, Johannesburg, South Africa

**Keywords:** Wives, Coping strategy, Men sexual health problems, Conjugal relationship

## Abstract

**Background:**

Systematic studies on the association between men’s sexual dysfunction (low sexual desire, ejaculation disorders, erectile dysfunctions, genital ulcers, testicular disorders, prostate cancer or sexually transmitted infections) and marital conflict are emerging. However, the coping strategies adopted by wives in such circumstances are not commonly reported in the literature. Male sexual functioning is vital to the marital relationship, lack of it can result in intolerable cohabitation or relationship breakdown, and could also cause infertility, infidelity, and arouse stigma in Nigeria. The understanding of coping strategies by female partners could guide in the counselling and treatment of men’s sexual health problems. Effective coping has the potential to lessen or prevent negative outcomes, and thereby could reduce marital conflict.

**Objectives:**

This study examined the coping strategies adopted by women whose husbands have reproductive health challenges in two of the five states with the highest proportion of divorce/separation in Nigeria.

**Methods:**

Four focus group discussions were conducted in two local government areas. The women were recruited from a quantitative couple-study for men with sexual health problems. Focus group responses were transcribed and analysed using systematic-content-analysis with thematic organisation of the summaries and systematic typologies of participants’ responses.

**Results:**

The results revealed the coping strategies employed by women in this environment: seeking guidance from their religious leaders and family doctors, physical-sexual-therapy, abstinence and concubinage. The participants indicated that they encountered difficulties in discussing their husbands’ sexual health problems with a third party.

**Conclusion:**

The study concludes that husband’s sexual ability is crucial to the sustenance of the marital relationship. Religious leaders and family doctors often serve as mediators to husband-wife conflict management. Counselling is recommended in cases of sexual health problems. Husbands should be encouraged to seek treatment and share their sexual challenges with their spouse. The medical officers and religious leaders could also be trained in family-conflict management.

**Electronic supplementary material:**

The online version of this article (doi:10.1186/s12978-017-0341-2) contains supplementary material, which is available to authorized users.

## Plain English summary

There are many male reproductive health challenges, such as low sexual desire, ejaculation disorders, penile disorders, impotence, and painful erections; these conditions may be associated with negative changes in intimate relationships including marital dissatisfaction and conflict. Male reproductive health challenges also include genital ulcers, testicular disorders, prostate cancer or sexually transmitted infections that could result in infertility or a breakdown of the intimate relationship. Couples facing infertility may experience shame, especially in traditional African settings where the importance of masculinity and patriarchy remain strong and childlessness is greatly stigmatized. Support for reproductive health can be critical to sustaining the marital relationship and the quality of this relationship.

This study assessed the coping strategies adopted by women whose husbands have reproductive health challenges in two Nigerian states with some of the highest rate of divorce. Participants were recruited from a quantitative couple-study where their male partners experienced sexual health problems. Four focus group discussions were organised in two local government areas. Respondents were asked about their knowledge of male sexual disorders, the challenges experienced by their husbands, the support they provided, their sex life, their current level of sexual satisfaction, and the strategies used to cope with their husband’s conditions.

The results of these discussions revealed that their husbands experienced a range of sexual disorders: low sexual desire, ejaculation disorder, impotence, erectile dysfunction, genital ulcers, testicular disorders and prostate cancer. It also found that participants sought guidance from community/religious leaders and family doctors, received physical-sexual-therapy, abstained from sexual relations and concubinage as strategies to cope with these conditions.

The coping strategies adopted by women could be important for marriage counsellors, social workers, and medical personnel. Counselling is recommended for the management of men’s sexual health problems, especially among women as a crucial tool to reduce marital conflict. Men should also be encouraged to seek treatment and to share their sexual challenges with their partners.

## Background

When the husband suffers from a sexual disease, both the marital partners are affected [1–3]. How the wife provides support and copes with these conditions is crucial to the quality of the relationship and its longevity [4]. Studies have examined spousal communication in cases of prostate cancer and sexually transmitted infections (STI) [5–7]. In addition, a number of studies have investigated coping behaviours of wives of alcoholics, marital and domestic conflict, where coping varied by culture [6–11]. However, studies that have documented the coping strategies that the wives employed in instances of husband’s sexual challenges in Nigeria, especially in terms of qualitative assessment, are missing in the literature.

Male reproductive health challenges are a range of disorders that affect the male reproductive system. These include penile disorders, erectile dysfunction, balanitis (swelling of the foreskin or penis), prostate cancer, genital ulcers, testicular disorders, low sperm count, painful or premature ejaculation, loss of libido, urethral discharge, and sexually transmitted infections [12–15]. These conditions, depending on their nature, if left untreated, could affect wellness [16, 17] with great consequences on the marital relationship [4, 16, 17] and disruptions in other areas of life [4, 16–19]. Although these conditions may not be life-threatening [15, 17], they are rarely reported [4, 20, 21] and are associated with social stigma [4, 15, 19], especially in Nigeria where discussions of sexual health are rare [17, 22].

There has been a global increase in male sexual disorders [4, 18, 20, 23, 24], and more than three out of every 10 Nigerian men have reported erectile dysfunction or another sexual problem [15, 17, 20–22, 25]. Although, the prevalence of these conditions are also high in other countries such as India, the mortality rate is higher in Nigeria [23, 26]. Sexual dysfunction threatens wellbeing and the future of their families [20, 21, 27]. It has implications for both men and their intimate partners [16, 20, 21]. In the traditional African system, where conjugal unions are sacred and expected to result in child bearing, strategies for sustaining such conjugal unions are essential [22, 28]. Specifically, husband-wife conflict affects the family relationship and has damaging social and economic consequences [4, 16, 19]. The incidence of marital instability, disruption and disintegration, and conflict resulting in violence or separation have increased worldwide, and in Nigeria [4, 20, 29]. While several factors have been suspected to be responsible for the husband-wife conflict, the impact of husband’s reproductive health problems have not been well documented [27, 30, 31].

This study is anchored on the way-of-coping theory, as developed by Folkman and Lazarus in 1980s. This theory defines coping as the sum of cognitive and behavioural efforts to handle demands (either internal or external) which are considered taxing, demanding or problematic [32]. In this regard, coping strategies are reactive behaviours employed to seek solutions or adapt to situations that emerge due to stressors [33] (i.e. husband sexual problem). While there are variants of coping strategy typologies [32–36], the simplified ideas are: altering the problem directly, altering one’s view of the problem, and managing the unpleasant feelings aroused by the problem [32, 36]. Similar understandings of coping have been used in other sexual health studies [4, 19, 37]. This study is concerned with the specific coping efforts, including behavioral actions of women in managing the sexual health problems of their husbands and the implications for their marriage.

Some instances of male sexual dysfunction are not preventable; however, the resulting marital conflict may have solution through effective management. To date, the coping methods of marital conflict or dissatisfaction caused by husband’s sexual problems have not been explored in the literature, especially in Nigeria. This study is, therefore timely, considering the increased rate of divorce, martial conflict and their associated consequences such as multiple sexual networking, ‘concubinage’ and vulnerability to HIV/AIDS and other STIs in sub-Saharan Africa [4, 38, 39]. The aim of this study is to describe the coping strategies of women confronted with reproductive health challenges in their husbands.

## Methods

### Study design

This study used focus group discussions (FGD) to explore the coping strategies adopted by women whose husbands had reproductive health disorders. Focus group discussions were used to encourage interactions among participants. The focus group discussions offered the opportunity to seek clarifications, raise follow-up questions and probe for more information.

Elements of framework analysis were used in data analysis. According to Ritchie and Spencer (1994) and Green and Thorogood (2004), framework analysis involves series of interconnected stages ranging from familiarisation, identifying a thematic framework, indexing, charting, mapping and interpretation [40, 41]. In addition, the technique permits the themes to evolve from the research questions as well as the responses from participants [40–43]. Framework analysis focuses on the development of real-life findings through the use of content analysis method in which responses were summarised and classified into themes [40, 41, 44]. Framework analysis is suited for applied research where the concern is to have practical approaches or offer solutions to a problem, especially on health-related issues.

### Study location

These focus groups were part of a larger study on male sexual diseases and relationships in Nigeria, funded by the Covenant University, Nigeria in 2011 (*Grant No: CUCRID-RG/AMOO/PHD/2007; details also indicated at the funding section*). The study was conducted in two states (Lagos and Osun), two of those with the highest rate of divorce/separation in the country [45]. In addition, the two states have a similar ethnic profile, and are bound by a common Yoruba language. One local government area (LGA) was selected in each state and a convenience sample of health facilities were then selected. Permissions were obtained from the management of each of the health facilities: two state hospitals, 19 private hospitals and eight traditional medical homes.

### Recruitment of participants

The parent study was a couple-study in which husband and wife were selected randomly on their clinic days and interviewed after informed consent had been obtained. Most men did not attend clinics with their wives, therefore, health personnel assisted in inviting the majority of male participants. Focus group participants were screened and recruited from the parent study among the wives of men who reported a current or past sexual disorder. An additional permission was obtained from the couple to participate in the FGD. Women under-15 and above 50 years of age were excluded, as women of reproductive age were the target-group of interest. In a marital relationship, we considered the intimate partners to be husband and wife, hence the choice of wives as the sample. The basic characteristics of participants (such as age, occupation, education, and so on) were assessed at the commencement of the interview.

### Data collection

A community hall was chosen for FGDs, where participants would feel comfortable, void of distractions, free to discuss, and easily accessible. Two FGDs were conducted per state with 8 to 12 participants each. The participants were split into two age groups (15–34 and 35–50 years), this was done to gain insight into variations in coping between age groups. For each of the age groups (15–34 and 35–50), only two FGDs were held. The small number of focus groups was due to a limited number of invitees available. Participants were volunteers and no incentive was provided due to limited funding. Nevertheless, the small number of FGDs was manageable and provided sufficient data. Each FGD lasted between 90 min and 2 h. The discussion continued until little or no new information was provided and theoretical saturation was reached.

A medical practitioner moderated the FGDs; they were chosen to avoid misinterpretation of health conditions. Discussions were held in Yoruba (local dialect), though some individuals combined Yoruba and English, or used Pidgin English. The focus group guide was adapted from the Golombok Rust Inventory of Sexual Satisfaction (GRISS), the Sexual Satisfaction Scale, the International Index of Erectile Function (IIEF-5) and the sexual function questionnaire [16, 19, 31, 46, 47]. Respondents were asked about their knowledge and experience of male sexual reproductive problems.

### Validity and integrity of the data

The wide range of participants’ characteristics (age, education, occupation and sexual problem) makes the findings more representative and aid in contrasting opinions among the participants. To ensure further validity and integrity of the data, texts were reviewed by participants to ensure correctness. The transcripts were also reviewed by researchers with qualitative expertise who were not involved in this study. Multiple readings of transcripts were conducted to detect further themes, to confirm that all transcripts follow the same verbatim approach to transcription and to reduce bias in the identification of themes [41]. The results of the analysis were also presented to non-participating women and colleagues who considered the suitability of the results.

### Data analysis

Field notes were taken during all focus groups. Responses were transcribed and analysed using ‘systematic-content-analysis’ [4, 40]. The transcripts and field notes were read several times to have an overall impression of the data. Recurrent responses and common themes were classified using the ‘scissors and paste’ approach [40].

Repeated review of transcript data and field notes eased identification of the emerging and recurrent concepts. Concepts were coded and organised into categories for each transcript and then merged together. Themes were further refined by adding more concepts, split or combined [4, 19, 41, 43, 44, 47, 48]. Responses that could not be directly grouped were discussed among reviewers and re-grouped. The relevance and importance of responses and the connections with other themes were discussed.

A Microsoft Word document was created detailing responses to each question, these were later cross-tabulated by age group for comparison: (1) 15–34 years and (2) 35–50 years. The results were benchmarked with existing literature. Data analysis adhered to qualitative research review guidelines (RATS) that places emphasis on the relevance of research questions, appropriateness of methods, transparency, and soundness of interpretive approach [40, 49]. The consolidated criteria for reporting qualitative research (COREQ) was used in presentation of this research [48].

## Results

The majority of participants were 35 years and older and were married for an average of 8 years. Many respondents were economically active; they were engaged in trading or paid employment, while a few were full-time housewives. Among those who had never attended school, one in five could not read or write, but discussions in the local dialect and pidgin language gave opportunity for full participation by all respondents. All participants were married; however, the number of marriages and age at first marriage were not discussed (Table 1).

**Table 1:** Background characteristics of participants in the focus group discussion

Selected characteristics	Total		Lagos State		Osun State	
	Frequency	%	Frequency	%	Frequency	%
Age group						
15–34	18	40.5	10	45.5	8	40.0
35–50	24	59.5	12	54.5	12	60.0
Total	42	100.0	22	100.0	20	100.0
Education attainment						
None	4	9.5	-	-	4	20.0
Primary education	16	38.1	8	36.4	8	40.0
Secondary education	18	42.9	11	50.0	7	35.0
Tertiary	4	9.5	3	13.6	1	5.0
Total	42	100.0	22	100	20	100
Religious affiliation						
Christianity	19	45.2	12	54.5	7	35.0
Islam	19	45.2	9	40.9	10	50.0
Others	4	9.5	1	4.5	3	15.0
Total	42	100.0	22	100.0	20	100.0
Occupation						
Full-time housewife	4	9.5	2	9.1	2	10.0
Farming/manual jobs	7	16.7	2	9.1	5	25.0
Trading	20	47.6	11	50.0	9	45.0
Clerical jobs/services	11	26.2	7	31.8	4	20.0
Total	42	100.0	22	100.0	20	100.0
Duration of marriage						
≤ 4 years	8	19.0	4	18.2	4	20.0
5–9 years	27	64.3	14	63.6	13	65.0
10 years & above	7	16.7	4	18.2	3	15.0
Total	42	100.0	22	100.0	20	100.0

Source: Authors’ computation

### Reproductive health challenges identified

Participants indicated the sexual problems experienced by their spouses. Few women used the correct medical terminology for the disorders described. Notwithstanding, experienced medical facilitators were able to appropriately classify the conditions described by the respondents. The most common reproductive health challenges described by participants were: low sexual desire, ejaculation disorders, penile disorders, impotence (“okobo”/ “akura”), erectile dysfunction, and genital ulcers (“egbo abẹ”). A small number of women also mentioned testicular disorders and prostate cancer (“jẹjẹrẹ”). A woman aged 15–34 expressed that *“After the surgery, he (*her husband*) had some time ago, things changed, ‘no action’ again”*. Another woman (35–50 years) pointed out that her husband had prostate cancer but it had been removed. Other sexual health problems experienced are described in the following excerpts:

*“Everything is ok for us, but the doctor confirmed that he has a challenge with the quantity of the sperm.*” (**Woman, aged 15–34**).

*“My husband has a problem with continuing erection, he sometimes withdraws midway” (* ***Woman*** **, aged 35–50).**

### Coping mechanisms

A number of coping strategies to prevent, stem conflict or decrease escalation to divorce/separation were described in the FGDs. These strategies were used to manage the consequences of the husband’s sexual problems. A popular response from participants was that sexual disorders and/or infidelity underlay most domestic quarrels, but that these could be curtailed if the wife appropriately managed the situation.

**Fig. 1:**
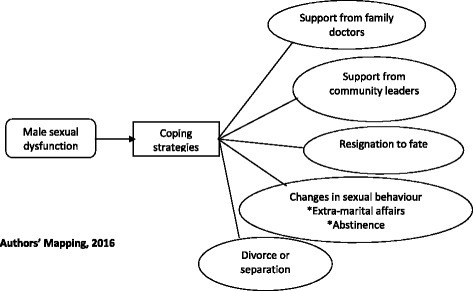
Primary themes from the focus group discussion on women coping strategies where husbands have reproductive health challenges

Primary themes and sub-themes of coping strategies included seeking support from family doctors, seeking support from community leaders, resignation to fate, changes in sexual behavior and an intention to divorce, as depicted in Fig. 1.

#### Support from family doctors

Across all discussions, seeking support from family doctors was one of the important steps in coping. Nearly all participants complained to their family doctors of the sexual health problems of their husbands. The general perception among these participants was that the medical doctors and other health personnel were *“divine helpers”* in family matters, especially as it relates to the husband-wife relationship and *“there is no reason to hide from them”*. Participants believed health personnel were trained to be discreet. Excerpts from a few of the participants in both age groups are as follows:

*“Why would you hide from those that can help you”? “You can’t hide from those who* [can] *deliver you” (* ***Woman, aged*** **35–50**).

*“Doctors are held in high esteem in our town, they are highly respected and most people have confidence in them because they are trained to keep secrets” (* ***W*** **oman aged 15–34).**

#### Resignation to fate

The results of the analysis revealed a popular coping strategy among the wives, a “resignation to fate approach”, where no effort was made to resolve the health problem, and the situation was considered closed or what they are destined to bear*.* Women describing this strategy were mostly older women, and the sexual problems ranged from ejaculation problems to low sperm count. Other comments supporting this response showed that “*keeping to oneself (on the issue) is the best option*”. A woman (aged 40) indicated that *“an issue like this is not openly discussed”,* hence the need to “*remain with it”* (to be silent over it)*.* However, another participant (in age 35–50) believed that *“If there is a deep affection between [the] couple, come rain and the sun, they must stay together”.* Almost one-quarter of all participants indicated they have been ‘holding-on to fate’ in managing these health concerns. Related to this, another woman (aged 35–50) considered a *‘silent posture’* over disagreements on sexual matters as the best option to sustain the marriage: *“for me, it is the best option for me, so as to allow tension to reduce”*.

#### Support from community leaders

The next strategy for coping with their husband’s sexual problems was to seek support from community leaders, such as religious leaders (e.g. pastors, imams). Women in this category had husbands with libido problems and in some cases believed that their husbands’ disorders were the result of a spiritual attack. Seven of every ten participants reported seeking support from their spiritual leaders. Although, responses were not recorded by religious affiliation, remarks were made by more than half of all participants.

In addition to community leaders, support was often sought from relatives. Discussions highlighted that the couple may also be invited by elders in the community. In this context, the husband or wife had the opportunity to express their grievances while the elders intervened to resolve conflict or offer advice. The participants reported that punishments were given to the erring party or the guilty party was warned. A woman, aged 47, reported that *“the fine could be anything: money, food or sacrifice”.* Another woman (age 30), indicated that *“it* [also] *depends on the ‘seriousness’ of the offence or the elders involved”*. This was supported by two women (aged 35–50 years), who stated that if it is a sexual matter, the husband will be ordered to have sex with his spouse, unless the husband is impotent. Another woman (age 35–50) indicated that, “*elders will know what to do*”. Participants described that every decision reached by the elders must be strictly adhered to.

#### Changes in sexual behaviour

The study further showed that wives adopted behavioural changes when faced with their husband’s sexual dysfunction. Results indicated that there were different strategies adopted in cases of sexual impotence; one out every five women adjusted their ‘sexual appetite’. Almost one-third of the respondents affirmed that they sought relationships outside marriage (concubinage or extra-marital affairs). Many could not imagine what marriage stands for without sex.

*“Now tell me, what is marriage without sex? Just to sleep together or be his cook?”* **(Woman, aged 15–34).**

Among those who adjusted their sexual behavior, were women whose husbands had sperm problems or those who could not engage in sex. The majority of women who opted for concubinage were those whose husbands had erection problems, many of them were 15–34 years. Two participants, however, believed that their situation did not warrant seeking external sexual partners. These participants believed there was hope, the condition may be temporary and, according to them, seeking help could resolve the problems.

Few women, aged 35–50, indicated that they have adopted abstinence as a solution. Three of the participating women stated that the situation gave them the opportunity to pursue businesses and work without distorting their marital relationship. These women were older and most had at least one child. Most of the women who claimed they had been abstaining from sex and those who indicated that they diverted their attention to their businesses and spiritual matters described ‘sexual intercourse as a non-primary issue’. A woman (aged 35–50) responded that *“after all, sex is not food”*. These women believed the situation gave them the privilege of focusing on other family issues, friends, and their careers. Another woman (aged 35–50) responded: *“we are only missing sexual closeness; we are not physically distant”.* Only two respondents asserted that they adopted what can be termed “physical sexual therapy intervention” to salvage their relationships. This, according to the respondents, comprised of foreplay and creating fun with their husbands.

#### Divorce or separation

Responses from younger women (aged 15–34 years) favoured separation if the problem persisted. A participant indicated that, “if your husband cannot have sex with you, *you will always be feeling you are not a woman or not attractive”.* While women in this group affirmed that it was negligence on the part of husband to allow such problems to progress untreated, they also indicated that *“there might not be any reason to “endure agony” arising from* [any] *man’s negligence”*. Another woman, aged 15–34, indicated that having children was crucial to marriage, and if the husband could not impregnate her, she would not hesitate to leave. An excerpt from another woman in the same age group shows:

*“If you want to manage it you can stay. […] If you want a* child *desperately, you don’t need to wait or announce your departure from his house”* **(Woman, aged 15–34)**.

Among women who preferred to stay married, they explained this choice based on the desire for children and economic reasons.

*“If your man is the only bread winner of the family, you would not want to divorce or leave him just because of that [sex]”* (**Woman, aged 15–34).**

*“If kids have been there before the problem comes, what do you do, would you leave your children? No, you just have to stay for the sake of your children” (* **Woman, aged 15–34).**

Few respondents believed that marriage becomes sacrosanct the moment it is solemnised and the party should stay together *“for better and for worst”*.

*“If there is deep loving affection between couple, come rain and the sun, they stay together” (* **Woman, aged 35–50).**

### Reporting sexual health problems

In addition to the primary themes identified, findings indicated that wives encountered challenges in reporting or discussing their husband’s sexual problem. The majority of participants indicated that when a problem is discovered; the wife cannot easily report their concerns. The general impression was that *“husbands are well-respected as the head, authority over the spouse and whatever issues pertaining to them (i.e. the husband) can only be uttered by them alone”*.

*“It is a painful situation”. “[…] It has never been a subject of open discussion since ages”.* **(Woman, aged 35–50).**

*“Most men you see around have so much under their clothes, I mean, they hide so many problems to themselves”* **(Woman, aged 35–50).**

There was consensus, especially among older participants, that sexual problems cannot be easily reported, not only due to tradition, but also because of the manner the husband may interpret this act. Few of their responses are as follows:

*“You remain silent over it”, “[…] it is not a subject of discussion with your neighbours”.* *(* **Woman, aged 35–50).**

*“If the man hears you tell anyone, he might scrape off your head” (* **Woman, aged 35–50).**

A few women indicated that it is dangerous to remain married to an impotent man: *“Coping with an impotent man is dangerous; they are always nervous, worry, aggressive and ill-tempered” (*Woman, aged 35–50).

Participants were asked whether they had recently discussed with their husbands the need to seek a solution. Almost half of the participants affirmed they have indirectly done so stating that *“action speaks louder than voice”*. Some suggestions offered by participants include, spiritual assistance, ‘mandating’ the family doctor to perform medical tests and reducing the consumption of certain food or alcohol.

## Discussion

This study provides evidence of coping strategies used by women whose husbands have sexual health problems. These findings extend beyond existing studies on husband-wife communication and wives coping with alcoholic spouses [5–7] or sexual function, attitude and lifestyles [9, 11, 16, 17, 20, 21]. Apart from adding to the body of knowledge on marital relationships, the findings may help in sustaining relationships notwithstanding the threat of sexual dysfunction, especially in the study location and by extension sub-Saharan Africa. While little research has focused on the intimate sexual relationship, this study exclusively dealt with the wife’s response to sexual challenges faced by her husband.

Among the important contributions of this study, is the development of a thematic framework demonstrating the coping strategies of women with husbands that have reproductive health problems. Several of the primary themes suggested that the conjugal relationship could be sustained in spite of these sexual health problems. It also highlighted that rather than resigning to fate alone, seeking guidance from health practitioners, especially family doctors and discussion between the spouses (husband-wife communication) could help. This finding is in concordance with coping mechanisms that suggest that appropriate responses could deal with external stressors in a manner to avoid conflict [32, 33, 35]. Access to health practitioners for the management of sexual health problems could prevent domestic conflict, as found in other studies [4–7]. Older respondents were more likely to be in favour of adjusting their sex life, such as abstinence and sexual therapy, rather than seeking external partners or separation.

The patterns of responses indicated two groups: (1) situations in which women may separate, divorce or engage in concubinage, and (2) situations in which women would endure sexual problems and maintain the marriage. The majority in the first group had husbands with erectile problem or suffered from premature ejaculation. Women in the second group had children and husbands with genital ulcers or testicular cancer. The second group also included women whose husbands were the breadwinners, women that resigned to fate or believed there was hope for the problem to be solved. The finding that women might not want to divorce or separate if children are involved, is similar to findings from Botha and Booysen (2012) and D’Souza, Karkada, Somayaji & Venkatesaperumal (2013). If the couple already had children, the wife might consider the future challenges children would face if they divorced [10, 28]. Many cited the presence of children and finances as fundamental factors for not divorcing. Additional information gathered suggested that financial buoyancy could play a vital role in keeping the marriage intact. The patriarchal system is strong in Nigeria, there are likely to be negative consequences for women who report their husband’s sexual health challenges [22].

A number of participants opted for separation or sexual relationships outside the marriage which may increase transmission of HIV/AIDS and other sexual transmitted infections [38, 39]. Since men rarely report sexual health problems [21], knowledge from their wives could be helpful to marriage counsellors, social workers, and medical personnel to guide diagnosis and management.

### Limitations of the study

There are limitations of this study, which include the use of a convenience sample of health facilities which limits generalizability of the findings. The sexual health problems were self-reported and were not confirmed through medical testing. The small number of FGDs could also be a limitation to the scope of the study. Finally, the combination of all male sexual health problems together may hide the variations inherent in different sexual challenges. This study only established responses by husband’s reproductive health problems and ages, other characteristics such as year of marriage were not collected.

## Conclusions

This study has added to the body of knowledge on coping strategies for sexual challenges. This study concludes that husband’s sexual health is crucial to the sustenance of the marital relationship and that religious leaders, as well as family doctors, may be indispensable mediators in husband-wife conflict management. However, the fact that the wives cannot easily discuss or report sexual health dysfunctions can be an obstacle to a healthy relationship. This work is essential for planning couple sexual health services in Nigeria, and where appropriate, couples should be encouraged to attend clinic together for counselling and treatment. Counselling on the management of men’s sexual health problems, especially among the wives, is recommended. Men should also be encouraged to seek treatment and share their sexual challenges with their partners (Additional file 1).

## Additional file

Additional file 1: Discussion guide on Male reproductive health challenges and wives coping strategies. (DOC 51 kb)

## Abbreviations

AIDS: Acquired Immunodeficiency Syndrome
COREQ: Consolidated criteria for reporting qualitative research
CUCRID: Covenant University Centre for Research, Innovation and Development
EAs: Enumeration areas
FGD: Focus group discussion
GRISS: Golombok rust inventory of sexual satisfaction
HIV: Human immunodeficiency virus
IIEF: International index of erectile function
LGA: Local government area
RATS: Qualitative Research Review Guidelines (Relevance of study question, appropriateness of methods, transparency, and soundness of interpretive approach)
STIs: Sexual transmitted infections
