# Supplementary material for: Male reproductive health challenges: appraisal of wives coping strategies
Source: Reprod Health. 2017 Jul 28;14:90. doi: 10.1186/s12978-017-0341-2 (PMC5534028; doi:10.1186/s12978-017-0341-2)
Supplement: Additional file 1: — Discussion guide on Male reproductive health challenges and wives coping strategies. (DOC 51 kb) [file 12978_2017_341_MOESM1_ESM.doc]

### Additional file

**Male Reproductive Health Challenges and Wives Coping Strategies**

### DISCUSSION GUIDE

## 1. PREAMBLES

### a. Salutation (Moderator)

I welcome everyone into this discussion forum on behalf of my colleagues here with me and myself. We appreciate your coming and we believe that the outcome of today’s discussions will be harnessed and serve as your valuable contributions to the wellbeing of human kinds.

- Acknowledge that the topic may seem sensitive or embarrassing; the participant should not worry. Moderator must alleviate the fear/worry and make it known to the participant that it is part of an academic exercise.
- Assure the participants about the confidentiality and anonymity of disseminated results.

### b. Background information

(i) Moderator is to introduce herself (name, affiliate), and the team members to the participants. (ii) Request the participants to introduce themselves one after the other. **Tell me about yourself**: Request to know: ***Age group, level of education, occupation, duration of marriage*** *(among others)****.*** The moderator should ask for clarification on any unclear information (where necessary).

### c. Golden Rules

**Moderator to read out and EXPLAIN the following guides: Certain rules that will guide this discussion are as follows:** *(1) Everyone is free to express her mind in this meeting; (2) There is No Right answer and there is No Wrong Answer; (3) Any participant is free to exit from this discussion at any time. (4) You are also free to skip any question you do not feel comfortable to answer. (5) All experiences and opinions are valid and very important.*

##

## 2. Moderator to introduce the topic of discussion

We have gathered at this morning / afternoon / evening to discuss a very important issue that relates to male sexual challenges and the strategies women are using to cope with the problems.

1. Let me now start by asking you what you understand by sexual challenges that men experience. What are the types that you know? **PROBE** for the definitions / understanding of various sexual health challenges as may be mentioned by each of the participants. Moderator to say: When you say …… (**repeat the problem/challenge mentioned)** what do you mean by it? Can you describe … (as mentioned by the participant)?

1. Consensus on definition/terminology for men sexual health problem. What specific word or terminology would you prefer us to (adopt) be using in discussing these group of challenges that everyone has just described now. Ask, is that term ok with you; what about you….? **Moderator to commence using respondents’ preferred term from this time onward.**

1. Now, can you tell us the specific sexual challenge(s) your spouse/partner is experiencing or ever experienced? **PROBE** whether the description is the same as indicated in the response to the first questions. If different, request for more explanation/description.
2. How did you know your husband/partner is having such disease(s)? How was it discovered? What were the worries you had when you discovered your husband has ….problem (**mention as applicable**)? Did you report or confide in anyone over the matter?
3. Have you had discussion with your husband about the need to seek a solution? **Yes/No. Probe for reasons to that answer. Ask for the specific support: Tell me the** specific support you gave to your spouse (or that you are currently giving him) in this respect. *Moderator should ask participant one after the other and probe for more details.*
4. Can I now ask: what were/are the strategies you used (or that you are using currently) to cope with your spouse’s current sexual health status? **Moderator to** ask each woman **what she has done or she is doing to prevent conflict, separation or divorce despite husband’s sexual health problem.** Probe for possible **SUPPORT/third party intervention**: medical, family or spiritual support, and so on). **Probe** for possible options available and why adopting a particular method.
5. Now, I will be calling each of the sexual challenges we have mentioned and ask us what coping method(s) are appropriate for such. **Moderator to say .…** What is the best coping method for …. (mention the problem). What about ….?

**Moderator may ask the Note Taker to remind her of other challenges earlier mentioned.**

1. **Participants (personal) perception of her sexual relationship with her husband.** How satisfy are you with your marriage presently? Would you describe your current sexual relationship with your spouse as satisfactory? Yes/No. **Probe for reasons to that answer. Moderator may ask:** How important is sex to you in your marriage.
2. What are the specific behavioural changes you have adopted due to sexual challenge of your husband? **Moderator may ask directly**: How is your sexual life currently? How many sexual partners do you have currently? PROBE for wife’s commitment to the marriage. Ask: How do you see your marriage in the next 3-5 years.
3. Do you have suggestion(s) for women who have or may have husbands that have these types of challenges? What do you think can be done to keep marriages despite these challenges?

- **ASK** if there is any query or question over all the discussion we just had.

**3. Appreciation and Closing**
